# Checkpoint CD24 function on tumor and immunotherapy

**DOI:** 10.3389/fimmu.2024.1367959

**Published:** 2024-02-29

**Authors:** Shiming Huang, Xiaobo Zhang, Yingtian Wei, Yueyong Xiao

**Affiliations:** ^1^ Department of Radiology, First Medical Center, Chinese People’s Liberation Army General Hospital, Beijing, China; ^2^ Graduate School, Chinese PLA Medical School, Beijing, China; ^3^ Department of Nuclear Medicine, Characteristic Medical Center of the Chinese People’s Armed Police Force, Tianjin, China

**Keywords:** CD24, immunotherapy, macrophage, Siglec-10, antibody

## Abstract

CD24 is a protein found on the surface of cells that plays a crucial role in the proliferation, invasion, and spread of cancer cells. It adheres to cell membranes through glycosylphosphatidylinositol (GPI) and is associated with the prognosis and survival rate of cancer patients. CD24 interacts with the inhibitory receptor Siglec-10 that is present on immune cells like natural killer cells and macrophages, leading to the inhibition of natural killer cell cytotoxicity and macrophage-mediated phagocytosis. This interaction helps tumor cells escape immune detection and attack. Although the use of CD24 as a immune checkpoint receptor target for cancer immunotherapy is still in its early stages, clinical trials have shown promising results. Monoclonal antibodies targeting CD24 have been found to be well-tolerated and safe. Other preclinical studies are exploring the use of chimeric antigen receptor (CAR) T cells, antibody-drug conjugates, and gene therapy to target CD24 and enhance the immune response against tumors. In summary, this review focuses on the role of CD24 in the immune system and provides evidence for CD24 as a promising immune checkpoint for cancer immunotherapy.

## 1 Introduction

The cluster of differentiation (CD) is a cell surface marker that functions as a receptor or ligand in cell signal transduction. The CD24 protein, which is encoded by the CD24 gene and serves as a cell surface marker in the immune system, facilitates the distinction between various immune cell types (1, 2). It plays an important role in regulating the activation and differentiation of immune cells such as B cells, T cells, natural killer cells, and dendrite cells (3). Furthermore, CD24 has been implicated in the pathogenesis of autoimmune diseases, including rheumatoid arthritis (4), as well as infectious diseases, such as tuberculosis (5).

Anti-tumor immunotherapy is becoming an effective strategy for treating tumors due to the development and clinical application of immune checkpoint drugs. These drugs target programmed cell death receptor 1 (PD-1) and its ligand PD-L1, as well as cytotoxic T lymphocyte associated protein 4 (CTLA 4). Recent research has shown that CD24, which is overexpressed on tumor cells, interacts with the inhibitory receptor Siglec-10 on macrophages (6). This interaction promotes immune escape of tumor cells and creates a new immune checkpoint in the form of a “don’t eat me” signal (7, 8). Therefore, CD24 has become a potential therapeutic target for cancer treatments, including antibody drugs, gene therapy, chimeric antigen receptor (CAR) T cell therapy, and more (9, 10). In addition, anti-tumor therapy targeting CD24 can also affect the tumor microenvironment and promote the immune system’s attack on the tumor, so CD24 may also be a target for immunotherapy (11). Therefore, this article will review the function of CD24 in immune system and its research progress in tumor immunotherapy.

## 2 Structure and expression of CD24

In 1990, the CD24 gene of humans was first cloned, homolog to the mouse CD24a (12). The gene sequence for CD24 can be found in at least five different locations on the human genome, specifically on chromosomes 1, 6, 15, 20, and Y (13). CD24 mRNA is transcribed from the chromosome 6q21 (14). The human CD24 cDNA has an open reading frame of 0.24 kb and a 3’ untranslated region of 1.8 kb. Additionally, a dinucleotide deletion in the 3’ untranslated region of CD24 can affect the stability of its mRNA (13).

CD24 is a protein in humans that is initially produced as a precursor protein containing 80 residues. After cleavage of both the C-terminal glycosylphosphatidylinositol (GPI) anchored sequences and N-terminal signal sequences, mature peptides with 32 residues are produced (15). CD24 contains four potential N-linked glycosylation sites and one or more O-linked glycosylation sites, which means that the molecular weight of CD24 can range from 20 to 70 kDa depending on the tissue or cell type (16). The degree of glycosylation of CD24 is highly variable and cell type-dependent. Highly glycosylated CD24 must be anchored to lipid rafts in the plasma membrane by GPI anchoring proteins.

Although CD24 does not have any secondary structure, it is expressed on the surface of various cell types, including hematopoietic cells (such as T cells, B cells, myeloid cells, dendritic cells, and macrophages) and non-hematopoietic cells (such as keratinocytes, muscle fibers, neurons, renal tubular epithelial cells) (17).

Additionally, CD24 is highly expressed in different types of normal tissues, particularly the thyroid (18), pancreas (19), and esophagus, where it plays an essential role in regulating the development and activation of cells. However, the expression of CD24 differs in various species. For example, CD24 is expressed in mouse red blood cells but not in human red blood cells. Therefore, targeting CD24 is safer and doesn’t produce the same blood-related adverse reactions as CD47.

Studies have shown that the levels of CD24 mRNA and protein vary according to the stage of cell development or maturation. CD24 is highly expressed in developing cells, but is almost absent in mature cell stages (15). CD24 is known as the B-cell differentiation antigen and is a highly variable glycosylated protein. It is continuously expressed in B cells, from the very early stages such as progenitor or precursor B cells, to mature B cells until B cells are stimulated by antigens and converted into plasma cells, which secrete antibodies (20). CD24 plays a role in the initiation of apoptosis in B cells by activating mitogen-activated protein kinase (MAPK) through lipid rafts. In contrast to B cells, most promyelocytes are CD24 negative, while mature myelocytes are CD24 positive, indicating a specific association with granulocyte maturation (21).

CD24 is a protein found on the surface of tumor cells that plays a crucial role in regulating the growth of various types of cancer, such as bladder cancer, liver cancer, B-cell lymphoma, prostate cancer, ovarian cancer, small cell and non-small cell lung cancer, breast cancer, and salivary gland cancer. Unlike CD47, which is also a protein that signals “don’t eat me” to immune cells, CD24 has limited distribution in healthy tissues but is highly expressed in tumor tissues. CD24 binds to different proteins on the surface of tumor cells, such as Siglec E, Siglec-10, L1 cell adhesion molecule, and platelet selectin, but only CD24/Siglec-10 is associated with phagocytic function (22). The expression of CD24 in tumor cells is regulated by various factors, including T cell factor-4, secreted frizzled related protein 1, non-coding RNAs, and hypoxia-inducible factors.

At present, we possess a great deal of knowledge regarding CD24 in B cells. However, we have comparatively less information about its existence in different species and its expression in other immune or non-immune cells. CD24 holds the structure of a typical cell adhesion molecule, which anchors lipid rafts through glycosyl inositol. It regulates signaling pathways both inside and outside cells and plays a key role in the adhesion between cells and stroma (23).

## 3 Function of CD24 in immune system

### 3.1 CD24 function in the adaptive immune system

CD24 was initially recognized as a marker for B cells. It is highly expressed in B cell progenitors but not in terminally differentiated plasma cells, as it is lost during the maturation of B cells (24). CD24 deficiency results in a decline in the number of immature B cells and advanced pre-B cells in the bone marrow. Activated B cell’s CD24 acts as a co-stimulator to boost the clonal expansion of CD4 T cells. CD24 triggers apoptosis in human B cells by transmitting signals through a glycolipid-enriched membrane (GEM) domain/raft. This signaling system includes linker for activation of T cells, Ras, Src family PTKs, and trimer G protein (25). When CD24 cross-links, it enhances the binding of CD24 to Lyn protein tyrosine kinase in GEM, and the activity of Lyn kinase is increased. Furthermore, the cross-linking of B cell receptor for Ag and CD24 has a synergistic effect on apoptosis induction. CD24 is responsible for mediating B cell apoptosis and empowering cancers with immune escape ability by directly inhibiting T cell proliferation (26).

Similarly, CD24 is highly expressed on immature T cells, but its expression decreases after T cell maturation. However, after T cell activation, CD24 expression rapidly upregulates. The main difference in the expression of CD24 between B cells and T cells is that CD24 expression upregulates on activated T cells. CD24 plays an essential role in the optimal activation and survival of specific T cells in the peripheral lymphatic organs and central nervous system (27).

In addition, the high expression of CD24 on tumor cells can inhibit TCR and BCR-related kinases through the binding of Siglec-10 on the surface of B cells and T cells. As a result, the activation of TCR and BCR is blocked, ultimately promoting tumor immune escape (28, 29).

### 3.2 CD24 function in the innate immune system

The main function of CD24 expressed in all innate immune cells (such as DCs and macrophages) is to present endogenous antigens. On microglia, CD24 helps in the proliferation and activation of pathogenic T cells (30). CD24 also plays a role in facilitating tumor progression by supporting the escape of tumor cells from macrophage-mediated phagocytosis through CD24/Siglec-10 signaling pathway (26). This provides a nutritional protective shield for the tumor cells. Additionally, the interaction between CD24 and high expression of Siglec-10 in NK cells mediates impaired NK cell function. However, it has been found that NK cells can selectively eradicate cells with lower differentiation levels, such as CD24+ ovarian cancer stem cells, by relying on the activation of Natural Killer Group 2D receptors (31).

In addition, the CD24 on DCs interacts with human Siglec-10 to negatively regulate the rapid homeostatic proliferation of T cells, the immune responses and host inflammation triggered by damage related molecules, and cause RNA viruses to evade host immunity (32). CD24+ DCs in lymph nodes can also promote the differentiation of virus antigen-specific T cells into effector T cells (33). In addition, CD24 binds to danger-associated molecular patterns and interacts with Siglec G on DCs to form a three-molecule complex on DCs, which can regulate the function of DCs.

CD24 has an important regulatory role in the function of antigen-presenting cells (APCs). The interaction between CD24 and Siglec-10 may be a significant pathway for CD24-mediated tumor immune evasion.

## 4 Biological function of CD24 in tumors

### 4.1 CD24 and immune evasion

CD24 has been found to exhibit high expression or amplification levels in various types of cancer stem cells and cancers such as gliomas, pancreatic cancer, retinoblastoma, cervical carcinoma, non-small-cell lung carcinoma, breast cancer, hepatocellular carcinoma, prostate cancer, renal cell carcinoma, urothelial carcinoma, pineal parenchymal tumors, and ovarian cancer (34). However, it is an exception in multiple myeloma, where the expression of CD24 is significantly down-regulated when compared to normal B-cell lines (35). This could be due to the fact that CD24 is expressed in pre-B lymphocytes, which is maintained in mature resting B cells, but downregulated during the maturation of plasma cells, which are terminally differentiated B cells.

In 2019, it was found that CD24 acts as a new “don’t eat me” signal for tumor cells to inhibit phagocytosis by macrophages in the innate immune system (36).

CD24 expression is upregulated in tumor cells, while Siglec-10 is expressed in macrophages of the tumor microenvironment (TME), which indicates a possible interaction between Siglec-10 and CD24. This interaction between CD24 and Siglec-10 is associated to the inhibition of macrophage-mediated phagocytosis, natural killer cytotoxicity, and evasion of the tumor immune system.

The phagocytosis of macrophages against tumors is regulated by a series of signals, including pro-phagocytosis signals (“ eat me “) and anti-phagocytosis signals (“ don’t eat me “). Many phagocytosis signals were expressed on the surface of tumor cells, including tumor-associated antigen, signal lymphocyte activation molecule family member 7, calreticulin and endoplasmic reticulum chaperone protein. However, there are also some anti-phagocytosis signals on the surface of tumor cells, including PD-L1, β2-microglobulin, CD47 and CD24 (37). These “don’t eat me” signals interact with receptors on the surface of macrophages, including Siglec-10, signal regulatory protein α (SIRPα), PD-1 and leukocyte immunoglobulin-like receptor-1.

It has been discovered that the ability of macrophages to phagocytic tumor cells is significantly improved by using monoclonal antibodies to block the connection between CD24 and the receptor or by silencing the CD24 gene. This improved phagocytic ability is positively correlated with the expression of Siglec10 (38). In conclusion, CD24 is believed to act as an anti-phagocytosis signaling protein that allows tumor cells to escape the immune system.

The interaction of CD24 and Siglec-10 triggers a cascade of immunosuppressive signals. After cytoplasmic tyrosine signaling is phosphorylated by Src family tyrosine kinases, Siglec-10 recruits and activates proteins containing the SH2 domain, specifically SHP-1, SHP-2 or suppressor of cytokine signaling 3 (SOCS3). As an important member of the tyrosine phosphatase family, SHP-1 can specifically bind to the phosphorylated tyrosine in the cellular immunoreceptor tyrosine-based inhibitory motif (ITIM) and catalyze its dephosphorylation (39). The absence of Siglec-10 or SHP-1 enhances the activation of NF-κB. Overexpression of CD24 promotes activation of NF-κB, while silencing of CD24 attenuates its activation in tumor cells. Overexpression of CD24 leads to activation of cell signaling proteins such as Akt, ERK, NF-κB and MMP-9 (40). In addition, intracellular signal transduction involving cytokines, growth factors, cell adhesion molecules and extracellular matrix can be negatively regulated.

### 4.2 CD24 and metastasis

CD24 is essential for the development of tumors. In some types of cancer, high levels of CD24 are associated with shorter survival times for patients. Studies have shown that reducing CD24 levels can trigger apoptosis and inhibit the proliferation of tumors, while increasing CD24 expression can promote tumor growth and metastasis (41). In tumors where CD24 is overexpressed, tumor cells become more invasive and the level of N-cadherin protein increases while the expression of E-cadherin decreases. Research shows that CD24 induces epithelial-mesenchymal transformation (EMT) in ovarian cancer and amplifies intracellular signals associated with cell growth via the PI3K/Akt and MAPK pathways (42). And overexpression of CD24 enhanced invasion and migration of glioma cells (43). Changes in CD24 expression on the surface of tumor cells are associated with a variety of carcinogenic signaling pathways, including HER2, Src/STAT3, WNT/β-catenin, MAPK, AKT/mTOR, EGFR, and mirNA-related pathways (44). And the expression of CD24 promotes tumor growth and metastasis in a dose-dependent manner (45).

Metastasis of tumor cells is a complex process that involves the ability of the tumor to bind to platelets in the bloodstream. Additionally, the ability of tumors to bind to endothelial cells may also contribute to metastasis. Once the tumor cells enter the bloodstream, they must attach themselves to the endothelial cells lining the walls of blood vessels. CD24, a ligand for P-selectin and an adhesion receptor in activated platelets and endothelial cells, is believed to play a key role in tumor metastasis (46). The ectopic expression of CD24 in tumor cells leads to increased proliferation rates and activation of α4β1 and α3β1 integrins, which facilitate binding to P-selectin, collagen, and laminin, thus promoting cell migration (47, 48). However, during metastasis, the interaction between CD24+ embryonal tumor cells and the somatic microenvironment, such as fibroblasts, endothelial cells, or immune cells, can lead to downregulation of CD24 expression (49).

Src is a type of non-receptor tyrosine kinase that plays a key role in regulating signal transduction of various membrane receptors. Its interaction with CD24 can lead to the promotion of tumor invasion and metastasis. Knocking down CD24 can result in the increase of the non-activated form of Src (Y527 phosphorylation) and a reduction in the activated form of Src (Y416 phosphorylation) (50).

CD24 can activate Src kinase, which in turn initiates other pathways that are related to tumor progression such as the tumor suppressor issue factor pathway inhibitor 2, a tumor suppressor protein. Additionally, it also activates integrins such as α3β1 and α4β1 integrins, and STAT3 cytoplasmic transcription factors. Activated integrins can enhance tumor cell adhesion to extracellular components such as laminin, collagen I and IV, and fibronectin, thereby promoting tumor metastasis (51).

The Wnt family is a group of glucose-secreting lipoproteins that regulate multiple signal transduction processes through the transcriptional coactivator β-catenin. When the β protein is activated, it can cause changes in the expression of Jun, Myc and cyclin D genes, which are involved in cell growth and cycle. Ahmad et al. (52) have confirmed that CD24 interacts with the Wnt pathway through the activation of β-catenin. Immunoprecipitation results show that CD24 can directly interact with β-catenin. In addition, studies have also shown that Notch and Wnt/β-catenin signaling pathways also play important roles in tumor cells that overexpress CD24 (53). Therefore, CD24 may promote the growth and proliferation of tumor cells by activating the Wnt signaling pathway.

CD24 also interacts with the epidermal growth factor receptor (EGFR) family proteins. These proteins are often overexpressed on the surface of different types of tumors, such as colon and breast cancer. Researches have shown that CD24 can effectively inhibit the internalization and degradation of EGFR, maintaining the expression of EGFR and its restriction to lipid rafts (54). CD24 also plays a role in tumor cell infiltration and metastasis through mechanisms related to E-selectin (55). CD24 is the target of the transcription factor hypoxia-inducible Factor-1α (HIF-1α), which is upregulated under hypoxic conditions, including during tumor growth and metastasis (56).

Moreover, CD24 is a protein that is involved in various cellular functions such as adhesion between cells and substrates, recognition, proliferation, movement, extension, and signal transduction. Additionally, CD24 is associated with the pathological grading and prognosis of tumors, which has significant implications for developing effective treatment plans for tumors. Targeting tumor stem cells can be a possible strategy for treating tumors.

## 5 Targeting CD24 for cancer immunotherapy

### 5.1 Monoclonal antibodies

A recent phase III clinical trial study has shown that the CD24Fc treatment is safe and well-tolerated for hospitalized COVID-19 patients. This treatment significantly accelerates clinical improvement, inhibits disease progression, and reduces hospitalization time for COVID-19 patients who require oxygen support. These findings suggest that the inflammatory responses to tissue damage may offer a viable treatment option for hospitalized COVID-19 patients (57). CD24Fc treatment weakens systemic inflammation, triggers NK and T cells to restore homeostasis, and reduces the co-expression of cytokines and network connectivity related to the severity and pathogenesis of COVID-19 (58). This indicates that the CD24 monoclonal antibody can inhibit the inflammatory response and restore immune homeostasis.

Patients who receive allogeneic hematopoietic stem cell transplantation from unrelated donors matched with human leukocyte antigen often experience acute graft-versus-host disease (GVHD). In a phase 2 multicenter study, researchers combined CD24Fc with tacrolimus and methotrexate to treat adult hematological malignancies. The survival rate for grade 3 to 4 acute graft-versus-host disease at day 180 of CD24Fc treatment was 96.2%, compared with 73.6% in the control group, and no dose-limiting toxicity was observed in CD24Fc treated subjects. These results indicate that CD24Fc can effectively prevent the occurrence of acute GVHD in adult hematological malignancies with good tolerance and safety (59).

Monoclonal antibodies are a type of treatment used to target unique or overexpressed antigens on cancer cells. Several monoclonal antibodies targeting CD24 have been investigated in preclinical experiments in a variety of tumor models (60). CD24 is a powerful anti-phagocytic “don’t eat me” signal that protects tumor cells from Siglec-10 secreting macrophages. CD24 mAb, which blocks CD24/Siglec-10 signaling, is considered as a novel innate immune checkpoint inhibitor. One such antibody, ALB9, has been shown to reduce lung metastasis and improve survival rates in patients with highly metastatic breast (61) and bladder cancers (62).

SWA11 monoclonal antibody (IgG2a anti-CD24 mAb) has high affinity and specificity for tumor cells expressing CD24. CD24 is internalized after binding with SWA11 and accompanied by changes in STAT3-dependent gene expression and Src phosphorylation, thereby affecting tumor cell proliferation, adhesion, invasion, and gene expression. Moreover, in addition to the direct effect on CD24-positive tumor cells, SWA11 can also trigger important immune response mechanisms such as ADCC or complement activation, and strongly affects the cytokine environment within tumors and increases macrophage infiltration into tumor tissue, which may help improve overall treatment effectiveness (63).

SWA11 has demonstrated the ability to decrease the proliferation of human pancreatic, ovarian, and lung cancer cells (63). It has also been found to slow down tumor growth in human colorectal cancer xenograft models formed by injecting HT29 cells to nude mice (64). Furthermore, SWA11 pretreatment has shown to enhance the effectiveness of gemcitabine, particularly by disrupting angiogenesis and promoting macrophage infiltration. SWA11 has been also proven to enhance the anti-tumor efficacy of various chemotherapy drugs, including 5-fluorouracil, oxaliplatin, doxorubicin, paclitaxel and irinotecan (64). Additionally, SWA11 antibody has been found to significantly improve the effectiveness of cisplatin in embryonal carcinoma cells (49).

Clone SN3 is another anti-CD24 monoclonal antibody that enhances macrophage phagocytosis of CD24+ tumor cells by blocking the CD24/Siglec-10 signaling pathway (“don’t eat me” signal). It has been showed that Clone SN3 promote phagocytosis of patient-derived CD24+ cell lines from ovarian and breast cancer, and it improve survival *in vivo* through macrophage-mediated tumor growth inhibition (36). In mantle‐cell lymphoma (MCL), clone SN3 can significantly promote the phagocytosis of tumor-associated phagocytes on tumor cells, and the phagocytosis rate is similar to that of anti-CD47 monoclonal antibody (65). In addition, SN3 also increases phagocytosis of MCL cell lines by M2-like macrophages. After the administration of clone SN3, phagocytosis is secondary to the loss of CD24 signaling and plays a pro-phagocytosis role in a CD24-dependent manner, rather than Fc-mediated opsonism.

IMM47 is a humanized monoclonal antibody targeting CD24, which can inhibits CD24/Siglec-10 interactions through macrophage antigen presentation and increases the release of NK cytokines. IMM47 can to directly stimulate the immune response of T cells by destroying the inhibitory CD24/Siglec-10 interaction, and also directly activate NK cells through ADCP and ADCC. IMM47 has been shown to effectively reduce the growth of breast cancer tumors, and when used in combination with anti-PD-1 antibodies, it has a synergistic effect. These findings suggest that IMM47 has great potential as a cancer immunotherapy, whether used alone or in combination with other treatments (66).

Sun F et al. (67) produced a new monoclonal antibody called G7mAb that targets CD24 using hybridoma technology. After humanized modification, hG7-BM3 has almost the same 3-D structure as the parental chimeric antibody, and it exhibits similar binding activity, affinity and specificity for CD24. The *in vitro* experiments show that hG7-BM3 has reduced immunogenicity and high stability, which makes it safe and stable in the human body. Moreover, it enhances the lysis of CD24+HCC cells mediated by NK cells and peripheral blood mononuclear cells (67).

To further improve the anti-tumor effect, CD24 antibody G7S was attached to the GoX- loaded nanospheres to form novel lysosome‐targeting chimeras (Nanosphere - AntiCD24). Tumor cells that overexpress CD24 can selectively take in this nanosphere and transport CD24 protein from the cell membrane to lysosomes for degradation. As a result, degradation of CD24 reduces immunosuppression of macrophages regulated by the CD24/Siglec-10 signaling pathway. In addition, GOx loaded in “Nanosphere Anti CD24” can target the release and sustained consumption of endogenous glucose in tumor cells for starvation therapy. This strategy can potentially overcome immune suppression during the effector phase in the tumor microenvironment and has shown promising synergistic therapeutic effects for HCC (68).

rG7S-MICA is a new type of bispecific monoclonal antibody fusion protein. It consists of a single chain antibody fragment (scFv) that targets the tumor-associated antigen CD24 and human MHC class I-related chain A (MICA). The scFv used in rG7S-MICA is derived from G7mAb and can specifically recognize tumor cells with high expression of CD24. MICA, on the other hand, is the primary immunoligand of the natural killer cell receptor NK group 2 member D (NKG2D) receptor found on NK cells. Therefore, rG7S-MICA induces NK cell-mediated cytotoxicity by recruiting NK cells to CD24+ tumor cells, which significantly improves the affinity and antibody-dependent cell-mediated cytotoxicity (ADCC) effect (69).

Bi-specific antibody cG7-MICA, formed by the fusion of chimeric anti-CD24 antibody with NKG2D ligand MICA, can also enhance the ADCC effect by activating NK cells and also bind to CD24. By recruiting NK cells to the tumor site, cG7-MICA increases the expression of CD107a on the surface of NK cells *in vitro* and activates them to release interferon γ (IFN-γ) and tumor necrosis factor α (TNF-α). As a result, the anti-tumor effect of sorafenib is enhanced (70).

In summary, antibody drugs targeting CD24 have shown great clinical application prospects. However, it should not be ignored that due to the expression of CD24 on the surface of B cells and the possible off-target effect, it may cause a certain degree of damage to B cells *in vivo* (71). Therefore, the potential toxic side effects of antibodies targeting CD24 on tumor patients cannot be ruled out at present.

### 5.2 Antibody–drug conjugates

Antibody drug conjugate (ADC) is a targeted drug delivery system that connect antibodies to active cytotoxic drugs. The use of antibodies to deliver cytotoxic drugs to tumor cells can improve therapeutic index and reduce systemic toxicity associated with the proportion of cytotoxic drugs. Compared to other cancer therapies, ADC is characterized by its high efficiency in inhibiting off-target toxicity by limiting the exposure of cytotoxicity to normal cells (72).

The binding of anti CD24 monoclonal antibody to deglycosylated ricin A-chain not only delayed tumor growth in a small cell lung cancer xenograft model, but also resulted in durable complete remission and increased survival in a Burkitt’s lymphoma mouse model (73).

Nitric oxide (NO) donors play an important role in tumor physiology and carcinogenesis. Low levels of NO can promote tumor growth, but high levels of NO can promote the inhibitory activity or cytotoxicity of carcinogenic cells. HL-2 includes an attachable maleimide terminal and a cleaved, targeted disulfide bond. The disulfide bond is stable in the blood and can rapidly release two molecules of NO. The NO conjugated antibody HN-01 was prepared by binding the NO donor HL-2 with G7mAb using a thioether bond. Sun F et al. (74) conjugated NO-donating HL-2 with G7mAb through a thioether bond to generate an antibody nitric oxide conjugate (ANC) similar to an ADC immunoconjugate, named HN-01. HN-01 has high efficiency internalization and significantly increases NO release in hepatic carcinoma cells. HN-01 induces tumor cell apoptosis and inhibits tumor growth in tumor bearing nude mice through antibody dependent co toxicity. HN-01 can also increase the NO level of tumor cells. In addition, HN-01 can facilitate the release of Cyt c into the cytoplasm, induce mitochondrial respiratory inhibition, and ultimately lead to cell death. Compared with the use of HL-2 or G7mAb alone, HN-01 significantly improves antiproliferative effect on CD24+ HCC in a dose-dependent manner. Furthermore, the cytotoxicity is partially attributed to the proapoptotic activity of NO donors. HN-01 can also significantly and durable inhibit the growth of xenograft tumors and significantly prolong survival time (74).

G7mAb-DOX is a combination of DOX and G7mAb, formed using the cross-linking agent GMBS. G7mAb has a lower immunogenicity response than the CD24 antigen, and it has a strong affinity for this antigen. This makes it beneficial for reducing the risk of chemotherapy. G7mAb-DOX is specifically captured and endocytosed by CD24+ tumor cells *in vitro*, with an average of 2 drug molecules per antibody. And G7mAb-DOX can inhibit tumor growth and prolong the survival of HCC with higher efficacy and less systemic toxicity compared to other treatments (75).

SWA11-ZZ-PE38 immunoconjugate produced by SWA11 armed with PE38 can selectively and effectively target CD24 antigen, and kill colorectal cancer cells by inhibiting EF2 and stopping protein synthesis *in vivo*. SWA11-ZZ-PE38 can also significantly reduce the IC50 value, thereby significantly reducing the use of monoclonal antibodies (by 106 times) and their potential side effects (76).

In addition, SWA11-DOX is a highly cytotoxic drug that rapidly internalizes into small-cell lung cancer cells by binding to its specific cell surface antigen CD24. SWA11-DOX is capable of inhibiting thymidine incorporation by 50%, with a selectivity 100 times higher than free doxorubicin (77). This indicates that targeted binding to the cell surface antigen CD24, rapid internalization, and effective release of doxorubicin from monoclonal antibodies are necessary conditions for the selective and potent function of SWA11-DOX.

### 5.3 CAR T cell therapy

CAR is a type of synthetic receptor that plays a crucial role in recognizing and eliminating tumor cells expressing specific target antigens. It works by reactivating lymphocytes, most commonly T lymphocytes. The binding of CAR to target antigens expressed on the cell surface is not dependent on MHC receptors, resulting in strong T cell activation and a powerful anti-tumor response. One of the most successful examples of CAR-T cell therapy involves targeting CD19 in the treatment of B-cell malignancies, which was approved by the US Food and Drug Administration in 2017 (78).

CAR T cell immunotherapy has proven effective in treating hematological malignancies. To target CD24 in solid tumors, more preclinical studies are using engineered immune cells for treatment. Anti-CD24 CAR T cell therapy has been successful in reducing tumor growth and metastasis of human pancreatic adenocarcinoma xenografts in mice (79). This treatment has also been effective in CD24 subclone-bearing tumors, suggesting that targeting pancreatic cancer stem cells with this method could be a feasible therapeutic strategy for pancreatic cancer. Bispecific BCMA-CD24 CAR-T cells have shown almost complete tumor clearance ability against multiple myeloma cells *in vitro* and *in vivo* (80). However, the antibody used by CD24 CAR-T cells was mouse scFv SWA11, which may cause allergic reactions.

Yang P et al. (81) utilized a humanized CD24 scFv (hG7-BM3) to develop CAR T cells that target CD24 (called 24BBz). *In vitro* testing showed that 24BBz exhibited antigen-specific activation and dose-dependent cytotoxicity on CD24+ BRCA tumor cells. However, 24BBz has almost no cytotoxicity on CD24 negative BRCA cells. In addition, 24BBz showed significant anti-tumor effects in T cell infiltration in CD24 positive TNBC xenografts and tumor tissues, with some T cells experiencing depletion. No major organ pathological damage was observed during the treatment process. This study indicates that CD24 specific CAR-T cells have strong anti-tumor activity and have potential application value in the treatment of TNBC.

Studies have shown that CD24 CAR-T can block the CD24-siglece-10 pathway. Activated CD24 CAR-T cells can release TNF-α and IFN-γ, which can promote the polarization of macrophages into an M1-like macrophage phenotype. Reversal of CD24+ myeloma cells can polarize C-X-C chemokine receptor type 4 (CXCR4) positive macrophages into an M2-like phenotype, resulting in the promotion of an immune response of phagocytosis and clearance of macrophages. In addition, the expression of macrophages and neutrophils increased in tumor cells, while the expression of B cells, natural killer (NK)/T cells and dendritic cells decreased. However, after CD24-CAR T treatment, macrophages decreased to 80% of the normal level, neutrophils further increased, B cells increased, and natural killer (NK)/T cells and dendritic cells further decreased (82).

CAR-T therapy has been successful in treating some B-cell leukemia or lymphoma. However, when it comes to treating solid tumors, there are limitations such as high toxic side effects, limited penetration of solid tumors, off-target effects and other problem (83). These limitations are likely to affect the use of CAR-T therapies that target CD24+ solid tumors. CD24 is not only present in tumor cells, but also in monocytes, granulocytes, B/T lymphocytes and other normal cells at varying levels. Therefore, it is important to explore ways to design CAR-T cells with high affinity for CD24 on the surface of tumor cells, while also reducing the risk of toxic side effects on normal cells such as B cells by optimizing its structure.

### 5.4 CAR NK cell therapy

NK cells, in addition to T cells, have been shown to exhibit effective and specific cytotoxicity against CD24+ patient-derived ovarian cancer cells and ovarian cancer cell lines (84). Söhngen C. (85) has evaluated the potential of CAR therapy against CD24 in NK cells as an immunotherapy option for CD24+ urological malignancies such as renal cell, urothelial, GCT, and prostate cancer. The treatment of urological tumor cells with CD24-CAR NK cells results in decreased cell viability and induction of apoptosis, particularly in CD24+ tumor cells.

Another approach is to use dendritic cells loaded with antibody-coated cancer cells that target different surface antigens (including CD24) to cross-present tumor antigens to CD8+ T cells, which can promotes T-cell-mediated cytotoxicity in ovarian cancer and melanoma cell lines (86). In a One Single Site Clinical Study, 36 patients with primary hepatocellular carcinoma who underwent surgical resection received adjuvant therapy with autologous transfusions of cytokine and dendritic cell induced CD24 peptide loaded natural CD3+ CD56+ type II killer T lymphocytes (87).

The clinical trial results indicate that the treatment protocol was well-tolerated, with a low incidence of adverse events. Specifically, the most frequently reported adverse event was a temporary fever (<grade 3), which was observed in 19% of patients. Furthermore, the study found that patients who received 2 and 4 treatments had 4-year survival rates of 47% and 53%, respectively.

### 5.5 Gene therapy

One of the limitations of administering monoclonal antibodies derived from rodents is the potential for eliciting an immune response in humans. Conversely, human monoclonal antibodies are fraught with difficulties in terms of production and cost. As a result, gene therapy targeting CD24 is currently an area of active research. Gene therapy represents a biological therapeutic modality that utilizes a vector to introduce foreign nucleic acids into target cells. This process enables the augmentation or reduction of gene expression and facilitates the correction or compensation of genetic defects and anomalies that underlie disease states (88).

Gene ablation of CD24 and Siglec-10 has been found to be an effective method for targeting tumor cells and enhancing macrophage phagocytosis. RNA interference technology (RNAi) is the most common clinical treatment for malignant tumors. Sagiv et al. (64) successfully inhibited the growth and metastasis of pancreatic and colorectal cancer cell lines by using small interfering RNA knockdown of CD24 expression. Additionally, down-regulation of CD24 delayed tumorigenicity in nude mice with human cancer cell lines.

Similarly, CD24 knockdown therapy with shRNA has been shown to be effective in ovarian cancer (89) and lung cancer after bone metastasis (90). Transfecting CD24 shRNA vector (CD24-SHRNA) to knock down CD24 expression can induce apoptosis and inhibit cell viability in SKOV3 cells (91). *In vivo*, administration of CD24 shRNA can exert a tumor suppressive effect by inducing tumor cell apoptosis, inhibiting tumor cell proliferation, and reducing microvascular density (91). When siRNA molecules targeting CD24 are added to the growth medium of epithelial cancer cell lines (such as prostate cancer and breast cancer), it results in a the low expression of CD24, leading to reduced cell growth, and changes in the actin cytoskeleton, resulting in exercise injury (92).

Therefore, knocking down CD24 expression may become an effective method for the future treatment of CD24+ tumors. Additionally, it may be possible to enhance the ability of macrophages to attack tumor cells by simultaneously silencing the gene expression of both CD47 and CD24 (93).

RNAi therapies have become more specific and selective over the years, but the potential for off-target effects and their effectiveness in treating tumor patients still need to be considered by researchers. These factors may restrict the future development and application of RNAi drugs that target CD24.

## 6 Conclusion

CD24 is a protein that is not yet fully understood, but it has become a promising target for diagnosing, treating, and predicting the prognosis of many types of tumors. Many tumors have been found to have high levels of CD24, and activating the CD24/Siglec-10 pathway has been shown to inhibit the function of cytotoxic T cells and phagocytosis mediated by macrophages, which promotes tumor immune evasion. CD24 is also involved in tumor cell migration and metastasis and has been identified as a prognostic marker for various types of cancers (35, 94, 95). Therefore, CD24 has attracted a lot of attention as a potential target for drug therapy against tumor cells or tumor stem cells. Several oncology clinical trials worldwide are currently testing the clinical efficacy of anti-CD24-based tumor therapy, and many more are registered on ClinicalTrials.gov (10).

CD24 protein has significant importance in the research of immune system and cancer. It helps us to understand how the immune system works and how cancer develops. Research on CD24 may also lead to new ideas and methods for treating related diseases. Despite its rapid progress as a tumor therapeutic target, its clinical application still faces several challenges. For instance, it is necessary to further validate its efficacy and safety, identify the right patient population and optimize the treatment plan. Identifying the optimal patient population for CD24 as a therapeutic target to achieve individualized treatment is a crucial direction for future research. Additionally, further researches are required to demonstrate the various functions and potential molecular mechanisms of CD24 in the pathogenesis of tumor.

## Author contributions

SH: Conceptualization, Validation, Writing – original draft, Writing – review & editing. XZ: Conceptualization, Validation, Writing – review & editing. YW: Supervision, Validation, Writing – review & editing. YX: Conceptualization, Supervision, Validation, Writing – review & editing.
